# Fresh Pomegranate Juice Decreases Fasting Serum Erythropoietin in Patients with Type 2 Diabetes

**DOI:** 10.1155/2019/1269341

**Published:** 2019-04-18

**Authors:** Saleem A. Banihani, Shuaibu M. Shuaibu, Belal A. Al-Husein, Seham S. Makahleh

**Affiliations:** ^1^Department of Medical Laboratory Sciences, Jordan University of Science and Technology, Irbid 22110, Jordan; ^2^Department of Clinical Pharmacy, Jordan University of Science and Technology, Irbid 22110, Jordan

## Abstract

Pomegranate juice (PJ) has been recognized to have various biological benefits in several pathological conditions. One such benefit is the augmentation of hemoglobin level and the number of erythrocytes in the human body. Here, we assessed the short-term effect of fresh PJ on the level of Erythropoietin (EPO) in patients with type 2 diabetes (T2D) compared to healthy individuals. Blood samples from 59 participants with T2D and 30 healthy individuals were collected after a 12-hour fast and 3 hours after administration of fresh PJ at 1.5 mL per kg body weight. Serum glucose was measured by standard method and commercially available chemiluminescent immunoassay kits were used to determine serum EPO concentration. Mean changes in serum EPO levels 3 hours after ingesting PJ and before the juice ingestion (EPO response to PJ) for both diabetic and healthy participants were -2.002 ± 0.541 vs. - 0.041 ± 0.214, respectively (P = 0.0087). This EPO response to PJ was found not to be correlated with age (P = 0.6622) and gender (P = 0.5354) for patients with T2D, while a negative correlation (P = 0.0183) between EPO response to PJ and fasting serum glucose concentrations was observed in these patients. In conclusion, fresh PJ reduced serum EPO level in patients with T2D, but not in healthy individuals, 3 hours after ingesting the juice. The EPO response to PJ was found to be negatively correlated with fasting serum glucose, but not with age and gender, of patients with T2D. This trial is registered with* ClinicalTrials.gov Identifier*. NCT03902288.

## 1. Introduction

Pomegranate juice (PJ) has a unique chemical composition rich in antioxidant polyphenols (e.g., tannins, anthocyanins, gallic acid, chlorogenic acid, ferulic acid, and coumaric acids) and flavonoids (e.g., quercetin, catechin, and phloridzin) [1, 2]. Several studies revealed that PJ has a strong antioxidant activity, which is, to some extent, greater than the well-known antioxidants in green tea and red wine [3, 4]. PJ consumption was found to be associated with various health benefits due to these dietary phytochemicals. Studies have shown that PJ has anti-inflammatory [5, 6], anticancer [7], anti-atherogenic [8], antihypertensive [9, 10], and antidiabetic effects [11]. Very recently, pomegranate polyphenols supplementation (equivalent to levels in approximately 8 oz of PJ) has been found to improve cognitive and functional recovery in ischemic stroke patients [12].

Actually, the well-recognized mechanism by which PJ induces its health benefits in various pathological conditions is by reducing oxidative stress, an imbalance between reactive oxygen species (ROS) and antioxidants for the favor of the former [13], and lipid peroxidation [1, 14]. This reduction may occur by enhancing the activity of certain antioxidant enzymes, neutralizing the generated ROS, and inducing metal chelation activity [1].

Erythropoietin (EPO), also called hemopoietin or hematopoietin, is a glycoprotein secreted from interstitial fibroblasts in the kidney in response to cellular hypoxia [15]. The main known function of EPO is the stimulation erythrocytes production (erythropoiesis) in the bone marrow [16]. EPO secreted approximately at 10 mU mL^−1^ is sufficient to compensate for the normal erythrocyte's turnover. On the other hand, in cases of cellular hypoxia such as anemia, blood EPO level can reach up to 10000 mU mL^−1^ [16].

The evidence above shows the various biological effects of PJ in several pathological conditions. Particularly, in 2017, the results from Manthou and co-workers showed that PJ enhances the level of hemoglobin and the number of erythrocytes in the human body [17], while the authors did not investigate the exact mechanisms behind such effects. Here, we assessed the short-term effect of fresh PJ on the level of EPO in patients with type 2 diabetes (T2D) compared to healthy individuals. We chose patients with T2D as these people may develop hypoxic cases such as anemia.

## 2. Materials and Methods

### 2.1. Subjects

Eighty-nine participants were included in this study. The study included two groups: patients with T2D and control group. Both groups were age-matched. Fifty-nine patients with symptomatic T2D (with fasting serum glucose between the ranges of 7.1-15.8 mmol L^−1^, according to criteria of American Diabetes Association (2018) [18]) were recruited from the Diabetes Clinic at King Abdullah University Hospital and the Ministry of Health Irbid Central Laboratory in the North of Jordan. The patients (25 males and 34 females) were between the ages of 37 and 60 years. The control group included 30 healthy subjects. They were assessed for glycemic condition and other associated chronic diseases to ensure their health status before including them in the study.

Participant's exclusion criteria included the presence of chronic renal or hepatic diseases, pregnancy, and other hormonal therapies. In addition to that, all subjects who ever used hypoglycemic agents, dietary hormonal stimulants, and cigarette smoking 12-hours before the treatment and sample collection were also excluded.

### 2.2. Ethical Considerations

All participants who expressed an interest in the study signed a form indicating they would like to be contacted by a member of the research staff to acquire information, administered with pomegranate juice and blood sample collection. During the recruitment and screening phases, subjects were informed of the study purpose, risks/benefits related to their participation, proposed testing, and the necessary time engagement. An informed consent form was provided for all participants to sign upon their compliance to participate in the study. The study was conducted in accordance with the Declaration of Helsinki, and the protocol was approved by the Ethics Committee of Jordan University of Science and Technology (*IRB-189/02012*).

### 2.3. Pomegranate Juice Preparation

Hand picking was used to obtain the pomegranate fruits from Kufr Soom valley farms located in North of Jordan. The fruits were collected, washed, and stored in a refrigerator for subsequent use. To prepare the pomegranate juice, the ripened fruits were first cut open to obtain the fresh seeds using a sharp and clean kitchen knife. A shallow slit was made at the top of the fruit and then cut all the way over the top of the rind. Three cut line sections were formed from the top to the bottom of the fruit to reveal the inner seeds. Pulling and breaking the fruit apart created large sections and any pieces of pith that are visible are removed to separate the seeds from the rind (peel) and pith.

When all the seeds have been removed, a mesh strainer was used to remove the floating pieces of pith from the top of the water in the bowl. The pomegranate seeds (arils) were then placed into a squeezing machine (Philips, Japan) to release their juice and mesh strainers were used to strain and extract the pure juice.

### 2.4. Intervention

The administered oral dosage (1.5 mL kg^−1^ of body weight) of pomegranate juice was selected on the basis of our pilot study where a significant reduction in the fasting serum glucose was observed after an approximate 3-hour time frame after juice administration. It was also chosen as it is expected (if at that same dose) to have an effect on the erythropoietin serum level of selected subjects. This may provide a dual bioactive function of pomegranate juice by decreasing serum glucose level as well as having an erythropoietic effect. Blood samples were drawn from subjects using plane tubes (anticoagulant-free tubes) in the morning after 12-hour fasting period (no caloric intake for at least 12 hours). Participant subjects were then given fresh pomegranate juice at 1.5 mL kg^−1^ of their body weight. Estimated 5-minute time frame was used for juice consumption to avoid variance. Blood samples were also collected in other plain tubes after 3 hours from the zero time of juice consumption. The subjects were restricted from food and other drinks except water within the zero time and third hour of juice ingestion. Directly after blood clotted, the serum was separated from the whole blood sample and then immediately stored at a temperature of -20°C for analysis.

### 2.5. Biochemical Assays

All serum glucose measurements were made using standard colorimetric enzymatic methods with Accent 200 kits (PZ Cormay S.A Diagnostics, Lomianki, Poland) and BS-200 Chemistry Analyzer (Shenzhen Mindray Bio-Medical Electronics Co. Ltd., ShenZhen, China). For serum erythropoietin measurement, commercially available chemiluminescent immunoassay kits (Access EPO Immunoassay System, Beckman Coulter, Inc, USA) were used. The glucose and erythropoietin concentrations in serum collected before ingesting the juice served as a control for each subject and values were compared to those recorded after ingesting the juice.

### 2.6. Statistical Analysis

All data were tabulated and expressed as mean ± standard error of the mean (S.E.M). Analyses of continuous variables between treatment groups to detect differences were conducted with the use of Student's* t*-test. Correlation between variables was calculated using Spearman's correlation test. The Statistical Package GraphPad Prism (version 6.0) Software (GraphPad Software, San Diego, CA, USA) was used for all statistical assessments.* P* values of less than 0.05 were considered significant.

## 3. Results

**Figure 1:**
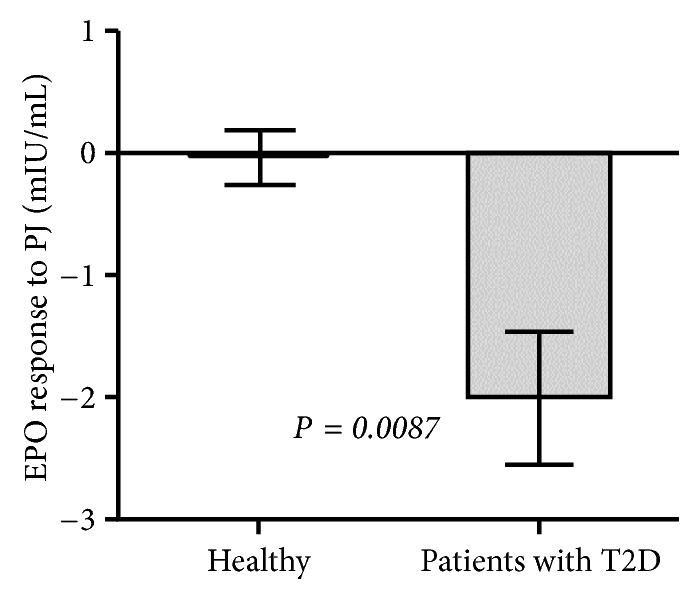
EPO response to PJ in T2D patients (n = 59) and in healthy individuals (n = 30). Values are given as the means ± S.E.M.

Figure 1 illustrates the EPO response to PJ in T2D patients (n = 59) and in healthy individuals (n = 30). EPO response to PJ represents the fasting serum concentration of EPO 3 hours after ingesting PJ (at 1.5 mL per kg body weight) minus the serum concentration of EPO before the juice ingestion. Mean EPO responses (change from baseline levels) to PJ for both diabetic and healthy participants were -2.002 ± 0.541 vs. - 0.041 ± 0.214, respectively; these means between both groups were found to be significantly different (*P =* 0.0087).

**Figure 2:**
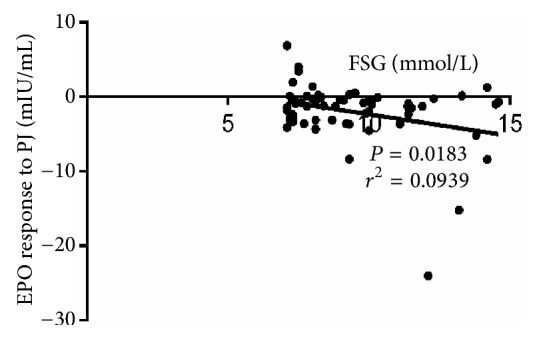
Correlation between EPO response to PJ and FSG concentrations in patients with T2D (n = 59).

Figure 2 demonstrates the correlation between EPO response to PJ and fasting serum glucose (FSG) concentrations for patients with T2D (n = 59) before the juice ingestion. As demonstrated in the figure, there was a negative correlation (*P* = 0.0183,* r*^2^ = 0.0939) between EPO response to PJ and FSG concentrations for patients with T2D.

**Figure 3:**
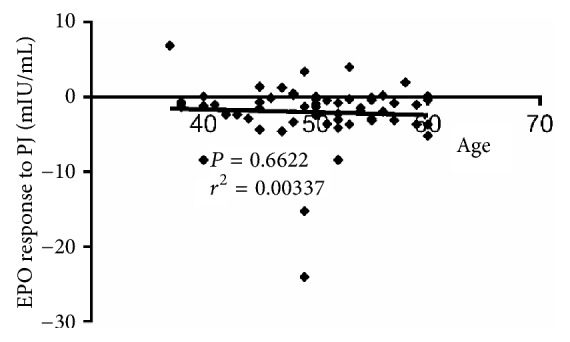
Correlation between the EPO response to PJ and age of patients with T2D (n = 59).

Figure 3 demonstrates the correlation between EPO response to PJ and age of patients with T2D (n = 59). As illustrated in this figure, no correlation (*P* = 0.6622,* r*^2^ = 0.00337) was found between the EPO response to PJ and the patients' age.

**Figure 4:**
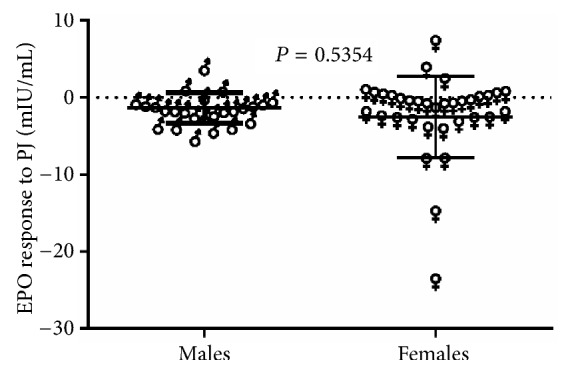
EPO response versus gender of patients with T2D (males, n = 25; females, n = 34).

Figure 4 demonstrates the EPO response to PJ versus gender of patients with T2D. As illustrated in the figure, no significant difference (*P* = 0.5354) in the mean values of EPO response to PJ was found between males (n = 25) and females (n = 34) with T2D.

## 4. Discussion

PJ has been recognized as having various health benefits in several pathological conditions [19, 20]. One of such benefits was enhancing hemoglobin and the number of erythrocytes in the human body [17]. In this work, for the first time, we asked whether fresh PJ has a direct effect on serum EPO concentration in patients with T2D and in healthy individuals. We recruited participants with T2D as these people may develop hypoxic cases such as anemia, given that EPO level is very crucial in such conditions. Unexpectedly, our results showed a negative EPO response to PJ (i.e., decrease in serum EPO concentration after ingesting PJ) in patients with T2D, but not in healthy individuals. In addition, EPO response to PJ was found to negatively correlate with FSG concentration, but not with age and gender, of patients with T2D.

In absence of anemia and other hypoxic cases, serum EPO levels in blood are quite low, at around 10 mU mL^−1^, while, in the presence of anemia or hypoxic stress, serum EPO levels may increase up to 1000-fold, reaching 10,000 mU mL^−1^ [16, 21]. Accordingly, in diabetic conditions, it is logical to find that the EPO response to PJ is higher in patients with T2D compared to healthy individuals. As well, it is logical to find that this negative EPO response is wider in patients of higher FSG levels, considering that the hypoxic stress is higher at advanced diabetic conditions [22, 23].

In 2011, Abe et al. found a significant positive correlation between EPO dose and homeostatic model assessment of insulin resistance (HOMA-IR) in patients with T2D, indicating a positive relationship between serum EPO and serum insulin in these patients [24]. In our previous study (2014), we have shown that fresh PJ at 1.5 mL/kg body weight decreases significantly the level of insulin 3 hours after drinking the juice in patients with T2D [11]. Accordingly, the decrease in insulin 3 hours after ingesting PJ could be a factor behind the observed negative EPO response to PJ.

Actually, we failed to underscore the exact factor behind the observed negative EPO response to PJ, given that PJ contains various bioactive compounds and each of which could be a suspected contributor in the mechanistic rout of EPO response and that these compounds are not yet directly linked with the bodily EPO levels. Besides, the mechanisms of anemia onset in T2D patients are multifactorial and are not yet well understood.

The previous findings by Manthou et al. (2017) showed that PJ consumption at 0.5 Liter/day for 14 days increased the red blood cell number and hemoglobin concentration in healthy subjects [17]. According to Manthaou and his co-workers such results may be due to increased erythropoiesis, which, subsequently, suggest an increase in EPO production; however, this suggestion was not supported by evaluating serum EPO level. Actually, even though these findings appear, to some extent, not in line with our findings, this difference has a strong justification. PJ contains antioxidants that would reduce oxidative stress and eventually lead to vasodilation of renal arteries and thus increase renal blood flow. This in turn might increase oxygenation of blood going to the kidneys and thus reduce EPO requirements. In our study we measured the direct EPO response to PJ among fasting individuals, used different PJ dose (1.5 mL/kg), and measured the serum EPO level 3 hours after ingesting PJ. In fact, in our study, the EPO response to PJ in healthy individuals was not noticeable maybe, at least in part, because the serum EPO range in healthy individuals is very narrow as we have explained above.

## 5. Conclusions

In conclusion, PJ, at 1.5 mL/kg body weight, reduced serum EPO level in patients with T2D, but not in healthy individuals, 3 hours after ingesting the juice. This EPO response to PJ was found to negatively correlate with FSG concentration, but not with age and gender, of patients with T2D.

Actually, it is worth mentioning that our laboratory is now designing a clinical study to measure the long-term effect of fresh pomegranate juice on EPO, particularly in patients with T2D, based on the encouraging findings from the current short-term study.

## Data Availability

The data used to support the findings of this study are available from the corresponding author upon request.
